# The Necessity of Diversity, Equity and Inclusion Training for Obstetrics and Gynecology: A Department-Wide Needs Assessment of an Academic Obstetrics and Gynecology Department

**DOI:** 10.1177/24731242251374427

**Published:** 2025-09-03

**Authors:** Ayana G.R. DeGaia, Shilpa Darivemula, Omar M. Young

**Affiliations:** ^1^ Department of Obstetrics & Gynecology, Harborview Medical Center, University of Washington, Seattle, Washington, USA.; ^2^ Division of Obstetrics, Gynecology, and Midwifery Department of Obstetrics and Gynecology University of North Carolina at Chapel Hill, Chapel Hill, North Carolina, USA.; ^3^ Division of Maternal-Fetal Medicine Department of Obstetrics & Gynecology, University of North Carolina at Chapel Hill School of Medicine, Chapel Hill, North Carolina, USA.

**Keywords:** DEI, obstetrics and gynecology, medical education, reproductive justice

## Abstract

**Introduction:**

The persistent disparities in the field of obstetrics and gynecology (OBGYN) have become increasingly visible in the public eye, while at the same time public discourse regarding the appropriateness and efficacy of diversity, equity and inclusion (DEI) education has become increasingly politically polarized.

**Background:**

While it has long been accepted that DEI training is an essential component of curricula offered in academic OBGYN departments, there remains a great deal of uncertainty regarding the best practices for developing and provisioning such training.

**Methods:**

In this article, the authors outline lessons learned from the process of developing an evidence-based department-wide needs assessment in order to evaluate the knowledge, attitudes, and behavior of members of a large academic OBGYN department, including an evaluation of the impact of prior DEI educational programming.

**Results:**

Of the 113 clinicians, nurses and administrative staff, the majority desired more DEI training and endorsed significant barriers to accessing it. There were also significant opportunities for improvement of health equity knowledge and clinical practice.

**Discussion:**

Although DEI training has been lauded for addressing health inequities, the findings of this needs assessment highlight existing gaps between desired and actual outcomes. It is essential that academic departments design, implement, and evaluate DEI strategies that are inclusive of the entire medical team, offer iterative and ongoing training opportunities, provide support for minoritized groups within the department, and involve a variety of stakeholders to improve our ability to provide equitable reproductive health care for all.

## Introduction

Over the past decade, there have been increasing efforts within academic obstetrics and gynecology (OBGYN) departments to increase diversity, equity and inclusion (DEI) educational programming, supported by increasing public outrage over worsening disparities in reproductive health and overwhelming scientific consensus that DEI activities are key to addressing them. However, the implementation of these activities has too often been limited in scope and approach, with little attention to the impact on the outcomes they are intended to improve. At a time when sociopolitical forces and the federal government are working tirelessly to dismantle DEI efforts and restrict reproductive health care nationwide, it is more important than ever that scientific efforts focus on producing evidence-based educational offerings to improve the provision of equitable and high-quality reproductive health care for all. In this study, we conducted a department-wide needs assessment of a single large academic OBGYN department to assess the equity-related knowledge, attitudes, and behaviors of its members in order to inform an evidence-based department-wide DEI curriculum.

## Background

Individual biases and systemic racism are key drivers of the pervasive inequities present in reproductive health, including within the field of OBGYN.^1–3^ It is well-known that Black and Native birthing people are 2–3 times as likely than their White counterparts to experience a pregnancy-related death and carry similarly disproportionate burdens of severe maternal morbidity as well as infant morbidity and mortality.^3^ In the wake of the Dobbs decision, state laws restricting access to abortion have been concentrated in states with the largest populations of Black birthing people, worsening maternal morbidity and mortality, reducing access to comprehensive reproductive health care, and threatening the bodily autonomy and safety of people seeking care for conditions such as miscarriage and stillbirth.^4,5^ And increasing restrictions on gender affirming care and even the existence of transgender and gender-nonconforming people are increasing stigma and creating barriers to accessing life-saving medical treatment.^6^

Persistent clinician biases perpetuating false beliefs about the biological differences between races and genders impact the treatment of conditions from pregnancy to endometriosis to abnormal uterine bleeding.^3^ And while biases and stereotypes engender clinician mistrust among minoritized communities seeking medical care, these impacts are also felt by clinicians from groups that are underrepresented in medicine (URMs).^3,7–11^ Mistreatment in the workplace affects most people of the global majority that enter the field of medicine, with studies reporting a high incidence of discrimination, harassment, humiliation, and biased evaluations by patients and colleagues.^11–13^ In narratives published in 2018 by Chescheir et al., a resident described being called “colored” by a nursing colleague, a foreign medical graduate struggled to be considered “good enough,” and a Black OBGYN was told by a flight attendant they were looking for an “actual physician” when she responded to an in-flight emergency call.^11,15^ Such negative experiences not only increase burnout, they contribute to a sense of hypervisibility and anxiety regarding the need to code-switch or center the comfort of white colleagues and supervisors, detract from any sense of belonging, and ultimately contribute to attrition and loss of URMs from academic medicine.^15^ In OBGYN specifically, these experiences may be contributing to the declining proportion of Black (10.2% to 7.9%), Hispanic (9.6% to 10.1%), and Native American or Alaska Native (0.2% to 0.1%) residents who entered the field from 2014 to 2019.^15^

With the increasing visibility of these disparities and the polarized tone of public discourse, there has been growing interest in the role of DEI initiatives within medical training. DEI education has widely come to be regarded as a foundational component of Graduate Medical Education (GME), a necessary step to begin to address professional and clinical disparities.^1^ In 2019, the Accreditation Council of Graduate Medical Education (ACGME) introduced a diversity accreditation standard mandating that all GME training programs include “practices that focus on…retention of a diverse and inclusive workforce”.^1–3^

In the aftermath of the 2020 Summer Uprising following the murder of George Floyd and the emergence of anti-Asian sentiment during the COVID-19 pandemic, there was a rapid increase in the number of academic OBGYN training programs that formalized DEI educational sessions into their residency curricula.^16^ These programs included a wide range of interventions, including didactics, skill-building, bystander training, morbidity and mortality conferences with a DEI component, and facilitated dialogues.^17,18^ Departments also introduced new DEI committees and leadership positions.^12,18^

Most publications evaluating these initiatives have found them to be extremely positive contributions to medical education, with most calling for expansion of activities and research in these areas. However, real gaps remain in both the implementation of these programs and their evaluation. Few programs have evaluated the impact of their programming on recruitment or retention of a diverse workforce, reduction in clinician bias or clinical decision-making, or improvement in clinical outcomes. A recent scoping review of DEI in GME programs found that the majority utilize a single, lecture-based DEI session to meet this ACGME requirement—an approach that is unlikely to have significant long-term impact.^1^ A 2021 national survey of OBGYN residents across the country described persistent, near-daily experiences of bias and racism impacting patients, trainees, and staff despite many having some exposure to DEI education.^7^

We believe that a key shortcoming of many DEI efforts in GME is their unilateral focus on the knowledge, attitudes, and behavior of resident physicians. To our knowledge, there are no studies assessing the entire departmental system: the adjunct staff, such as our nursing colleagues and front desk teams, along with faculty and trainee physicians. While there is collective consensus on the value of a team-based approach to patient care, our educational interventions to improve equity have often targeted trainees alone; trainees who repeatedly describe a sense of powerlessness when it comes to advocating for change without full departmental support.^7^ Excluding other departmental members from educational opportunities to improve DEI may contribute to the persistence of inequities in our field. Unfortunately, there is a lack of evidence-based best practices for the design of interdisciplinary, intradepartmental DEI curricula in academic OBGYN.

The purpose of this study is to evaluate the knowledge, attitudes, and behavior of members of an academic OBGYN department, to assess the impact of prior DEI educational programming, and to inform the design of an evidence-based DEI curriculum for OBGYN residents, faculty, and staff.

## Methods

This was a prospective cross-sectional study taking place from January 2023 to April 2023. A computer-based survey was adapted from a validated tool published by Person et al. in 2015, the Diversity Engagement Survey (DES), which is designed to measure DEI in academic medical environments.^9^ Questions were developed to address each of the eight inclusion factors described in the DES (Table 1), with responses tabulated on a 5-point Likert scale.^9^ In addition, a series of knowledge and behavior questions were crafted to evaluate respondents’ understanding of key health disparities and application of this knowledge to clinical practice. Finally, respondents were asked about their participation in DEI educational activities over the past 2 years.

**Table 1.** Needs assessment survey items by domain

	Definition	Response type	Survey items
Diversity Engagement Survey (DES) domains			
1. Common purpose	The individual experiences a connection to the mission, vision and values of the organization.	Five-point Likert scale	i. I feel connected and integral to the mission, vision and values of our department. ii. I want to learn how best to support my colleagues and create an inclusive and equitable workplace for people of diverse backgrounds. iii. The diversity of staff in our department is important to me and to our success as a department. iv. I want to learn more about how to reduce health disparities in OBGYN. v. There has already been a lot of focus on diversity, equity and inclusion and I think our department is already doing the best that it can.
2. Trust	The individual has confidence that the policies, practices, and procedures of the organization will allow them to bring their best and full self to work.	Five-point Likert scale	i. The policies, practices and procedures of our department allow me to bring my best and full self to work. ii. I worry there are individuals in our department who are disadvantaged due to their expressed identities or physical attributes.
3. Appreciation of individual attributes	The individual is valued and can successfully navigate the organizational structure in their expressed group identity.	Five-point Likert scale	i. My individuality is valued by my department. ii. I think there are individuals in our department who experience overt or hidden biases due to their expressed identities or physical attributes (inversely scored).
4. Sense of belonging	The individual experiences their social group identity being connected and accepted in the organization.	Five-point Likert scale	i. My expressed group identities (i.e., race, gender, religion, professional background, experiences) are valued within my department. ii. My department is welcoming to me and people like me.
5. Access to opportunity	The individual is able to find and utilize support for their professional development and advancement.	Five-point Likert scale	i. All members of our department have equal access to support for their professional development and advancement.
6. Equitable reward/recognition	The individual perceives the organization as having equitable compensation practices and nonfinancial incentives.	Five-point Likert scale	i. Our department compensates our team members in an equitable manner.
7. Cultural competence	The individual believes (they and their) institution has the capacity to make creative use of its diverse workforce in a way that meets business goals and enhances performance.	Five-point Likert scale	i. I can confidently differentiate among race, ethnicity, nationality and culture. ii. I can confidently differentiate among sex, gender identity, gender expression and sexual orientation. iii. I can define racism and describe its impacts on the health of individuals and populations in the US. iv. I can describe key health disparities in perinatal and neonatal health locally in [this state]. v. I can describe key health disparities in perinatal and neonatal health in the US. vi. I can describe key health disparities in gynecology locally in [this state]. vii. I can describe key health disparities in gynecology in the US. viii. I can describe evidence-based strategies for identifying implicit biases in the provision of care. ix. During clinical encounters, I am able to identify racist or discriminatory policies or actions. x. During clinical encounters, I am able to safely and adequately address situations involving racism or bias. xi. I can describe biases impacting contraceptive counseling and inequities around LARCs and surgical sterilization. xii. I am aware of how local political changes impact OBGYN care for my patients. xiii. I am aware of how national political changes impact OBGYN care for my patients.
8. Respect	The individual experiences a culture of civility and positive regard for diverse perspectives and ways of knowing.	Five-point Likert scale	i. In our department, we co-create a culture of civility and positive regard for diverse perspectives and ways of knowing.
Knowledge questions			
	—	True/False	i. (This state’s) WIC covers baby formula and menstrual products. ii. Gender identity is each person’s internal and individual experience of gender. iii. Hemorrhage is the number one cause of maternal death in North Carolina. iv. Ethnicity includes self-claimed or subjective identity and social behaviors linked to a perception of shared ancestry, nationality, history, cultural origins and possibly religion.
Behavior questions			
	—	Yes/No	i. I regularly ask my prenatal patients about access and barriers to healthy foods when counseling about obesity and weight gain in pregnancy. ii. I regularly ask patients about social support as a part of my pre-operative counseling. iii. I regularly ask my patients about postpartum bleeding expectations if I see them on L&D. iv. I regularly ask my patients about their access to menstrual products if they have menstrual cycles.

Requests for survey completion were sent via departmental email listservs to faculty physicians, midwives, advanced practice clinicians, nurses, medical assistants, administrative staff, and trainees within a single academic OBGYN department. Respondent demographics were collected, including self-identified race (Bureau of Labor Statistics categorization, other), ethnicity (Latinx/Hispanic, Non-Latinx/Hispanic, or other), and gender identity. Race and ethnicity data were collected because it was hypothesized that respondents from minoritized groups may have different responses due to their lived experience of racism. As recommended by Sullivan et al. in 2013, analyses of Likert scales questions were performed after grouping related questions into the predefined eight categories and then calculating a mean score for each category.^19^ Otherwise, standard descriptive statistics were used for analysis. This study was IRB exempt (IRB #22-2910).

## Results

One hundred thirteen (113) responses were obtained with an overall response rate of 27.5%. Of those responses, 19% (*n* = 22) were from faculty physicians, 3% from midwifery faculty (*n* = 3), 12% from trainees (*n* = 13), and the remainder from the nursing and office staff (Table 2). The majority of respondents self-identified as White (52%), Non-Latinx/Hispanic (58%), and cis-gender women (59%), which is reflective of the national makeup of academic OBGYN programs (Table 2).

**Table 2.** Demographics of survey respondents

	Frequency (%)
Age	
20–29 years	12 (11)
30–39 years	24 (21)
40–49 years	22 (19)
50–59 years	16 (14)
≥60 years	5 (5)
Race	
Asian	6 (5)
Black/African American	12 (11)
Native Alaska/American Indian	1 (1)
Pacific-Islander/Native Hawaiian	1 (1)
White	59 (52)
Other	7 (6)
Ethnic Group	
Latinx/Hispanic	10 (9)
Non-Latinx/Hispanic	65 (58)
Other	8 (7)
Nationality	
American	81 (72)
Other	5 (4)
Gender	
Cisgender woman	67 (59)
Cisgender man	8 (7)
Transgender woman	0 (0)
Transgender man	0 (0)
Nonbinary and/or gender nonconforming	1 (1)
Other	8 (7)

Most participants in our survey endorsed a shared sense of purpose in the DEI work within the department (84%). A majority endorsed the belief that the department co-creates a culture of civility and positive regard for diversity (69%), highlighting an alignment of values across the department and a desire to work towards equity. However, far fewer endorsed trust in the inclusivity of the department (43%), stating that not all members of the department are equitably supported, promoted, or recognized for their work (47%).

Respondents indicated a strong confidence in their abilities to identify and address inequities in their field (70%). However, reported practice patterns did not reflect understanding of key disparities. For example, only 23% of respondents endorsed routinely discussing access to healthy foods during prenatal visits. Gaps in OBGYN-specific social determinants and inequities were also noted. For example, many respondents mistakenly believed that the Special Supplemental Nutrition Program for Women, Infant, and Children covered menstrual supplies (40%).

Nearly one quarter of respondents endorsed having had no DEI training in the past 2 years, while 77% reported fewer than 5 hours of such training. Among those respondents who had never had DEI training before, 70% desired interprofessional DEI education. Eighty-three percent of those respondents that endorsed participating in DEI training in the past two years desired further training in DEI education. When asked which trainings they had attended, 38% of respondents had attended one of two departmental DEI activities—the DEI Grand Rounds series or the resident-led DEI lecture series. Notably, the latter offering was discontinued prior to data collection after the graduation of the resident physician who led and implemented this series.

When asked why DEI training is challenging to access, a majority endorsed lack of time (28%), restraints related to the format or timing of training (18%), and competing responsibilities (29%) as major barriers.

## Discussion

### Shared Sense of Purpose in DEI Work Despite Persistent Challenges

It is greatly encouraging to see that the majority of those surveyed shared the sense that DEI work was important to them and to the excellence of the department. That said, respondents also shared several challenges and barriers to participating in educational offerings.

### Lack of Access to Departmental DEI Education Despite Overwhelming Interest

Among respondents, barriers related to lack of time and formatting of training were commonly cited, reflecting the challenges experienced in all OBGYN departments balancing a host of clinical and administrative responsibilities with educational needs. For example, it may be difficult for outpatient clinic staff to block out an hour for DEI training rather than seeing patients. In systems and organizations where resources are limited and staffing shortages are omnipresent, ensuring attendance at DEI trainings requires support from senior leadership through the allocation of protected time to allow attendance, plans to minimize impact on clinical flow, longitudinal evaluation to ensure quality and relevance of trainings, and financial support to offer a wide range of DEI educational opportunities for faculty and trainees. This finding also highlights the importance of incorporating feedback from departmental stakeholders when planning the timing and format of DEI training. Changes to departmental compensation practices and administrative timetables could address both competing responsibilities and lack of dedicated time.

Strategies for increasing access to DEI education are numerous, including integration into ongoing educational, administrative, and clinical activities. Multiple studies have recommended inclusion of health equity training into existing departmental meetings as a practical way to arrange DEI education.^18,20^ Quality improvement activities can also serve as a pivotal opportunity to address disparate care and provide educational opportunities. One such example at our institution was the integration of the previously segregated resident clinic, which served a largely indigent population, with the more private-practice private-payer-predominant attending clinics. Not only was this a huge step toward ensuring more equitable and inclusive care for our diverse patient population, but it presented learning opportunities for clinicians and staff by increasing the diversity of the patients they cared for, better preparing trainees for lifelong practice by ensuring that their patient demographics were more reflective of the diversity they’d encounter upon graduation, and allowing seasoned faculty to apply their prodigious skill to a wider variety of clinical cases.

While these efforts were initially led by a small number of residents and staff, the success of this transition would not have been possible without the leadership and championing of our department chair. Removing this internal barrier to equitable care required significant buy-in from executive leadership and took about 10 months to implement.^21^ The successful transition to integrated clinics resulted from department leadership not only actively listening to equity concerns raised by all department stakeholders but also navigating the complexity of institutions, finances, and departmental needs to ensure this change occurred. This change offers hope for imaginative and practical DEI work within our discipline and serves to highlight the need for the involvement of members at all levels of an organization, especially leadership, for the longevity and applicability of future strategies seeking to mitigate structural and systemic inequities.

### Gaps in Knowledge and Practice Despite Confidence in DEI Knowledge

Our findings highlight a frequently observed discrepancy between respondents’ perceived understanding of key inequities in OBGYN care and their application of this knowledge to clinical practice. These results raise concerns about the effectiveness and scope of prior DEI education, including the topics covered, as well as the integration of the practices of cultural humility and critical self-inquiry. The practice of self-reflection is an essential component of DEI work, and education offerings providing instruction in this area or quality improvement measures that create time for this practice are extremely valuable.

Too often, the faceless nature of clinical care provision can lead to team members forgetting our shared goal of providing equitable care. A recent quality measure implemented to deconstruct this on our labor and delivery unit is a structured post-event debrief, in which all team members gather after a challenging experience to talk through the case in an organized manner. The guide for this debrief was co-created by a variety of stakeholders on the unit. The penultimate question asks for reflections on the event, including how systemic factors or bias may have contributed. This moment to face each other and to acknowledge the fear, hurt, concern, and rage about inequitable outcomes allows for humanization for all team members involved. And creating a safe space to acknowledge these and suggest repairs for individuals involved or structural changes to prevent future incidents has been extremely important. Effective facilitation of these debriefs is essential, but the resulting team trust and actionable steps to improve care on the unit are well worth it.

### The Cost to Trainees and Junior Faculty from Minoritized Groups

A 2023 review of foundational and aspirational strategies to improve DEI in GME highlighted the need to support underrepresented and historically marginalized physicians and to compensate faculty and residents for their DEI work.^16^ This last recommendation is especially pertinent in our study, as the most widely attended DEI educational offering attended by our respondents was designed and implemented by a Black resident physician, who was not compensated for her time and whose curriculum ended abruptly after her graduation. Ideally, this role would ultimately have been taken over by a faculty member or administrator with an appointed DEI role, appropriately protected time and compensation, and adequate resources to support more longitudinal efforts. However, the appointment of such a leader is often limited by interdepartmental, institutional, and political actors opposed to DEI programming.

In this setting, clinicians from racial and ethnic backgrounds historically URM often assume responsibility for the work they know needs to get done at a net detriment to their career interests or personal well-being. Campbell and Rodriguez highlight the term “minority tax” in academic medicine, in which URMs are burdened with additional diversity recruitment efforts, health equity work, or mentorship of URMs coming behind them without commiserate value placed upon that work for the purposes of reputation or promotion.^20^ While largely informal, many URM faculty endorse a sense of additional burden for teaching others about one’s lived experiences and becoming the voice representing all DEI work in the department without appropriate compensation, which can be a huge drain on physicians’ energy, time, and spirit. This syphoning can draw energy away from other professional pursuits, not to mention necessary self-care activities needed to excel in one of the most trying periods of a physician’s life. At a time when many URMs are facing increasing public scrutiny as to the legitimacy of their skills, accomplishments, and positions, it can also mean drawing disproportionate negative attention and even legal action from governmental agencies opposed to DEI programming in our current sociopolitical landscape. Wherever possible, it is essential that department and university leadership take a protective stance of their URM faculty and trainees, as well as the DEI work being done in their institutions to improve access to excellent education as well as equitable reproductive care for all.

### Health Equity Implications

Despite recent efforts to utilize DEI curricula to address reproductive health inequities, this department-wide needs assessment highlights existing gaps and opportunities for future work. While this study is limited by its cross-sectional and single-center design, we believe that our findings likely reflect the challenges faced by academic OBGYN departments around the country. Departments should continue to work to design, implement, and evaluate DEI strategies that are inclusive of the entire medical team. Fortunately, despite ongoing negative public discourse, those who have experienced DEI training desire more of it and for it to be as interprofessional as possible. The intentional formation of Health Equity divisions within departments of OBGYN that strive to represent the variety of experiences of all stakeholders of the department with particular care to flatten hierarchical power structures, oversee continuous DEI quality improvement and education offerings, and center the success and support of URMs will be crucial to this work to be successful and sustainable.^22^ This integrated representation can shed new light on complex challenges, unveil solutions that would otherwise be overlooked, improve collaboration, and break down the invisible yet ever-present power differentials within an academic department. Providing support and protection for URMs and others in our field who are doing the work, and involving a variety of members of the department at every level, will be essential to catalyzing powerful change and systemic improvements in reproductive health in the United States. During a political era where the very term “DEI” and any related activities are being indiscriminately targeted, it is more necessary than ever for our field to demonstrate moral courage by continuing this critical work and avoid anticipatory obedience to reprimands or consequences we fear may be coming. Only by centering from the margins will we be able to address the greatest reproductive health challenges of our time and improve reproductive health outcomes for all.

## Abbreviations Used

ACGME: Accreditation Council of Graduate Medical Education
DEI: Diversity, equity and inclusion
DES: Diversity Engagement Survey
GME: Graduate Medical Education
IRB: Institutional Review Board
OBGYN: Obstetrics and gynecology
URM: Underrepresented in medicine

## References

[1] Chung AS, Cardell A, Desai S, et al. Educational outcomes of diversity curricula in graduate medical education. J Grad Med Educ 2023;15(2):152–170; doi: 10.4300/JGME-D-22-00497.1 37139216 PMC10150806

[2] Swanson ML, Whetstone S, Illangasekare T, et al. Obstetrics and gynecology and reparations: The debt we owe (and continue to accumulate). Health Equity 2021;5(1):353–355; doi: 10.1089/heq.2021.0015 34084987 PMC8170721

[3] Howell EA, Zeitlin J. Improving hospital quality to reduce disparities in severe maternal morbidity and mortality. Semin Perinatol 2017;41(5):266–272; doi: 10.1053/j.semperi.2017.04.002 28735811 PMC5592149

[4] Kheyfets A, Dhaurali S, Feyock P, et al. The impact of hostile abortion legislation on the United States maternal mortality crisis: A call for increased abortion education. [published correction appears in Front Public Health]. Front Public Health 2024;12:1358617; doi: 10.3389/fpubh.2024.1358617. Front Public Health. 2023;11:1291668. Published 2023 Dec 5; doi: 10.3389/fpubh.2023.1291668 38371242 PMC10870982

[5] Brindis CD, Laitner MH, Clayton EW, et al. Health-care workforce implications of the Dobbs v Jackson Women’s Health Organization decision. Lancet 2024;403(10445):2747–2750; doi: 10.1016/S0140-6736(24)00581-6 38795713

[6] Quint M, Bailar S, Miranda A, et al. The AFFIRM Framework for gender-affirming care: Qualitative findings from the transgender and gender diverse health Equity Study. BMC Public Health 2025;25(1):491; doi: 10.1186/s12889-024-21261-7 39915834 PMC11800641

[7] Committee on Health Care for Underserved Women. ACOG Committee Opinion No. 729: Importance of social determinants of health and cultural awareness in the delivery of reproductive health care. Obstet Gynecol 2018;131(1):e43–e48; doi: 10.1097/AOG.0000000000002459 29266079

[8] Finkbeiner C, Doria C, Ellis-Kahana J, et al. Changing obstetrics and gynecology residency education to combat reproductive injustice: A call to action. Obstet Gynecol 2021;137(4):717–722; doi: 10.1097/AOG.0000000000004297 33706356

[9] Person SD, Jordan CG, Allison JJ, et al. Measuring diversity and inclusion in academic medicine: The Diversity Engagement Survey. Acad Med 2015;90(12):1675–1683; doi: 10.1097/ACM.0000000000000921 26466376 PMC5823241

[10] Cheng MY, Neves SL, Rainwater J, et al. Exploration of mistreatment and burnout among resident physicians: A cross-specialty Observational Study. Med Sci Educ 2020;30(1):315–321; doi: 10.1007/s40670-019-00905-z 34457673 PMC8368104

[11] Chescheir NC, Benner RS. Bias in obstetrician-gynecologists’ workplaces. Obstet Gynecol 2018;132(4):813–819; doi: 10.1097/AOG.0000000000002869 30204693

[12] Avery MD, Jennings JC, Germano E, et al. Interprofessional education between midwifery students and obstetrics and gynecology residents: An American College of Nurse-Midwives and American College of Obstetricians and Gynecologists collaboration. J Midwifery Womens Health 2020;65(2):257–264; doi: 10.1111/jmwh.13057 31965745 PMC7187383

[13] Campbell KM, Rodríguez JE. Addressing the minority tax: Perspectives from two diversity leaders on building minority faculty success in academic medicine. Acad Med 2019;94(12):1854–1857; doi: 10.1097/ACM.0000000000002839 31192803

[15] Nguyen BT, Mitchell-Chadwick N, Heyrana KJ. Declines in the proportion of US Black obstetrics and gynecology residents. JAMA Netw Open 2021;4(5):e219710; doi: 10.1001/jamanetworkopen.2021.9710 34009355

[14] Nguyen BT, Mitchell-Chadwick N, Heyrana KJ. Declines in the proportion of US Black obstetrics and gynecology residents. JAMA Netw Open 2021;4(5):e219710; doi: 10.1001/jamanetworkopen.2021.9710 34009355

[16] Boatright D, London M, Soriano AJ, et al. Strategies and best practices to improve diversity, equity, and inclusion among US graduate medical education programs. JAMA Netw Open 2023;6(2):e2255110; doi: 10.1001/jamanetworkopen.2022.55110 36753279 PMC9909494

[17] Hill Weller L, Rubinsky AD, Shade SB, et al. Lessons learned from implementing a diversity, equity, and inclusion curriculum for health research professionals at a large academic research institution. J Clin Transl Sci 2024;8(1):e22; doi: 10.1017/cts.2024.6 38384906 PMC10879992

[18] Reisinger-Kindle K, Dethier D, Wang V, et al. Health equity morbidity and mortality conferences in obstetrics and gynecology. Obstet Gynecol 2021;138(6):918–923; doi: 10.1097/AOG.0000000000004575 34735374

[19] Sullivan GM, Artino AR. Jr., Analyzing and interpreting data from likert-type scales. J Grad Med Educ 2013;5(4):541–542; doi: 10.4300/JGME-5-4-18 24454995 PMC3886444

[20] Moreu G, Isenberg N, Brauer M. How to promote diversity and inclusion in educational settings: Behavior change, climate surveys, and effective pro-diversity initiatives. Front Educ 2021;6; doi: 10.3389/feduc.2021.668250

[21] McClurg AB, Arora KS, Schiff LD, et al. Dismantling structural barriers: Resident clinics refocused on equity. Obstet Gynecol 2022;140(5):739–742; doi: 10.1097/AOG.0000000000004920 36201760

